# Disability pension receipt five years after cancer diagnosis: a matched registry study of working-age cancer survivors in Norway

**DOI:** 10.3389/frhs.2026.1913581

**Published:** 2026-09-25

**Authors:** Shahram Moradi, Steffen Torp, Roy Nielsen, Eliva Atieno Ambugo

**Affiliations:** 1 Research group for disability and inclusion, Department of Health, Social and Welfare Studies, University of South-Eastern Norway, Porsgrunn, Norway; 2 Research Group for Health Promotion in Settings, Department of Health, Social and Welfare Studies, University of South-Eastern Norway, Tønsberg, Norway; 3 Fafo Institute for Labor and Social Research, Oslo, Norway; 4 University College of Norwegian Correctional Service, Lillestrøm, Norway

**Keywords:** cancer, cancer stage, cancer survivors, disability pension, welfare state

## Abstract

**Objective:**

To examine receipt of partial or full disability pension (DP) five years after cancer diagnosis among working-age cancer survivors (CSs) in Norway and the associations of cancer-related and socioeconomic factors with DP receipt.

**Methods:**

Nationwide registry data included 11,188 individuals aged 30–55 diagnosed with first primary invasive cancer (2004–2008), all employed at diagnosis. CSs were matched to cancer-free controls at a 1:1 ratio based on age, sex, education, and employment at baseline. Conditional logistic regression estimated the association between cancer status and DP receipt. Additional analyses among CSs examined cancer type, cancer stage, and socioeconomic factors.

**Results:**

CSs had more than twice the odds of receiving DP compared with controls. Among CSs, advanced cancer stage and cancer type (e.g., respiratory, blood, and CNS-related cancers) were associated with higher odds of receiving DP, while skin cancer was associated with lower odds (compared to digestive organ cancers). Older age, lower education, lower income, and being unmarried were all associated with higher odds of receiving DP. Having young children (aged 0–5) versus none was associated with lower odds of DP, and no differences were observed by immigrant background.

**Conclusion:**

CSs had higher odds of receiving DP, particularly those with advanced cancer stage and certain cancer types. Socioeconomic disadvantages such as lower education or limited income were also associated with higher odds of receiving DP. These findings highlight the need for tailored occupational rehabilitation strategies that address both clinical and socioeconomic circumstances.

## Introduction

Advances in early detection and treatment have led to a growing population of working-age cancer survivors (CSs). While many return to work, long-term physical, psychological, and social consequences can impair their work ability (1). A major concern is the increased risk of disability pension (DP) receipt among CSs. DP is intended for individuals whose earning capacity has been permanently reduced because of illness or disability and may be awarded as either a partial or full benefit (2). Receipt of partial DP is compatible with continued paid employment, and DP receipt therefore does not necessarily indicate complete withdrawal from the workforce. Nevertheless, the long-term reduction in earning capacity and work participation associated with DP receipt may have economic and psychosocial consequences, including reduced income, lower retirement benefits, loss of professional identity, social isolation, and poorer mental well-being (3). Unlike sickness absence, DP generally reflects a more lasting reduction in earning capacity, making receipt of partial or full DP an important outcome for occupational rehabilitation policy.

In Norway, disability pension was administered by the Norwegian Labour and Welfare Administration (NAV) during the study period and was generally granted when illness or disability had permanently reduced an individual's earning capacity by at least 50%, following appropriate treatment and vocational rehabilitation efforts (4). Under the rules applicable during our follow-up period, recipients could combine disability pension with a limited amount of employment income. However, earnings above the permitted level could lead to reassessment of the individual's disability degree and a reduction in pension entitlement (4). The disability pension reform implemented in 2015 introduced a more gradual income-adjustment system, allowing recipients to increase their employment income without reassessment of the established disability degree (4). Because all five-year outcomes in the present study were measured between 2009 and 2013, the findings reflect the disability pension system in place before the 2015 reform.

Although several Nordic studies have examined DP receipt among survivors of specific cancer types, national-level evidence that includes all major cancer diagnoses remains limited. These studies consistently show that working-age CSs have higher odds of receiving DP compared to cancer-free counterparts. In Norway, studies have shown that CSs with breast and colorectal cancer have higher odds of DP, especially when diagnosed at advanced stages (5, 6). In Sweden, registry-based and longitudinal studies found higher odds of receiving DP among CSs relative to controls (7–9). In Denmark, similar patterns have been shown across several cancer types, with higher odds of DP receipt, particularly among CSs with blood or breast cancers (10, 11).

Cancer stage has consistently been associated with higher odds of DP receipt among CSs in prior studies, with those diagnosed at regional or distant stages more likely to receive DP due to more intensive treatment and greater functional impairments (5–7, 9). In addition, survivorship-related symptoms such as comorbid physical diseases, fatigue, cognitive problems, and emotional distress, may reduce work ability and be associated with higher odds of DP receipt (12–14).

Sociodemographic and work-related factors are also associated with DP receipt among CSs. Nordic studies have found that women, older individuals, and those with lower income or lower education have higher odds of receiving DP (15, 16). CSs living alone or with an immigrant background also have higher odds of receiving DP (17–19). Furthermore, CSs in manual or self-employed occupations have been found to have a higher likelihood of DP receipt (15, 16).

Because earlier studies have primarily examined single cancer diagnoses, evidence from Norway that covers all cancer types and examines associations between clinical and socioeconomic factors and DP receipt remains limited. In Norway, where the welfare system aims to support labor force participation, identifying factors associated with DP receipt among CSs is important for designing occupational rehabilitation and work-retention services.

We assessed receipt of partial or full DP five years after diagnosis because this follow-up period has been used in comparable Nordic registry-based studies of DP following cancer diagnosis (19, 20). Although DP may be granted at any time after diagnosis, assessment at five years captures longer-term DP receipt beyond the period of diagnosis and initial cancer treatment. Five years was also the longest follow-up period available consistently across participants in the parent CanWork dataset. Accordingly, the present study examines DP receipt among working-age CSs in Norway five years after diagnosis. First, we compare the receipt of partial or full DP at 5 years after cancer diagnosis between CSs and their matched peers without cancer. Second, we examine which clinical factors (cancer type and cancer stage) and sociodemographic factors (e.g., income and marital status) are associated with the odds of DP receipt among CSs.

## Methods

### Study design and data sources

This study is part of the broader Cancer and Work (CanWork) research project (21), which investigates factors influencing employment outcomes among CSs using linked registry data from the Cancer Registry of Norway and Statistics Norway's FD-Trygd database. A related CanWork publication used the same parent cohort and registry data sources to examine employment five years after cancer diagnosis among female CSs and matched cancer-free controls, with immigrant background categorized as native, Western immigrant, or non-Western immigrant (22). In contrast, the present study includes both women and men and focuses on receipt of partial or full disability pension rather than employment status. It also examines associations with cancer type, cancer stage, and sociodemographic and employment-related factors. Although the two studies were derived from the same parent cohort, they used study-specific analytic samples, outcomes, and analytical approaches.

The initial CanWork dataset comprised 58,683 individuals, including 19,561 CSs and 39,122 cancer-free controls initially selected at a 1:2 ratio. Figure 1 presents the derivation of the analytic sample. From the initial sample of 58,683 individuals, 5,031 died between baseline (T0) and the five-year follow-up (T5), including 4,661 CSs and 370 controls. These individuals were excluded before the study-specific 1:1 CEM. Thus, matching was conducted among CSs and controls who survived to T5. A further 289 individuals were excluded because of loss to follow-up, unregistered status, or out-migration. Among the 53,363 individuals remaining at this stage, 4,832 had missing values on one or more study measures. Missingness occurred in baseline marital status, income, employment industry, and employment status, as well as cancer stage/metastatic status among CSs. These exclusions reflected system-missing values, meaning that no usable value was recorded in the source data. They were distinct from valid registry-coded categories indicating unknown, unspecified, or unclassifiable information for employment industry, cancer stage, or cancer type. These registry-coded categories were retained as separate analytical categories rather than treated as missing data. The exclusions due to missing data comprised 1,543 of 14,796 CSs (10.4%) and 3,289 of 38,567 controls (8.5%), indicating that missingness was somewhat more common among CSs. An additional 290 duplicate records among controls and 7,433 individuals who were not employed at T0 were excluded. This resulted in 40,808 eligible individuals, comprising 11,203 CSs and 29,605 controls.

**Figure 1:**
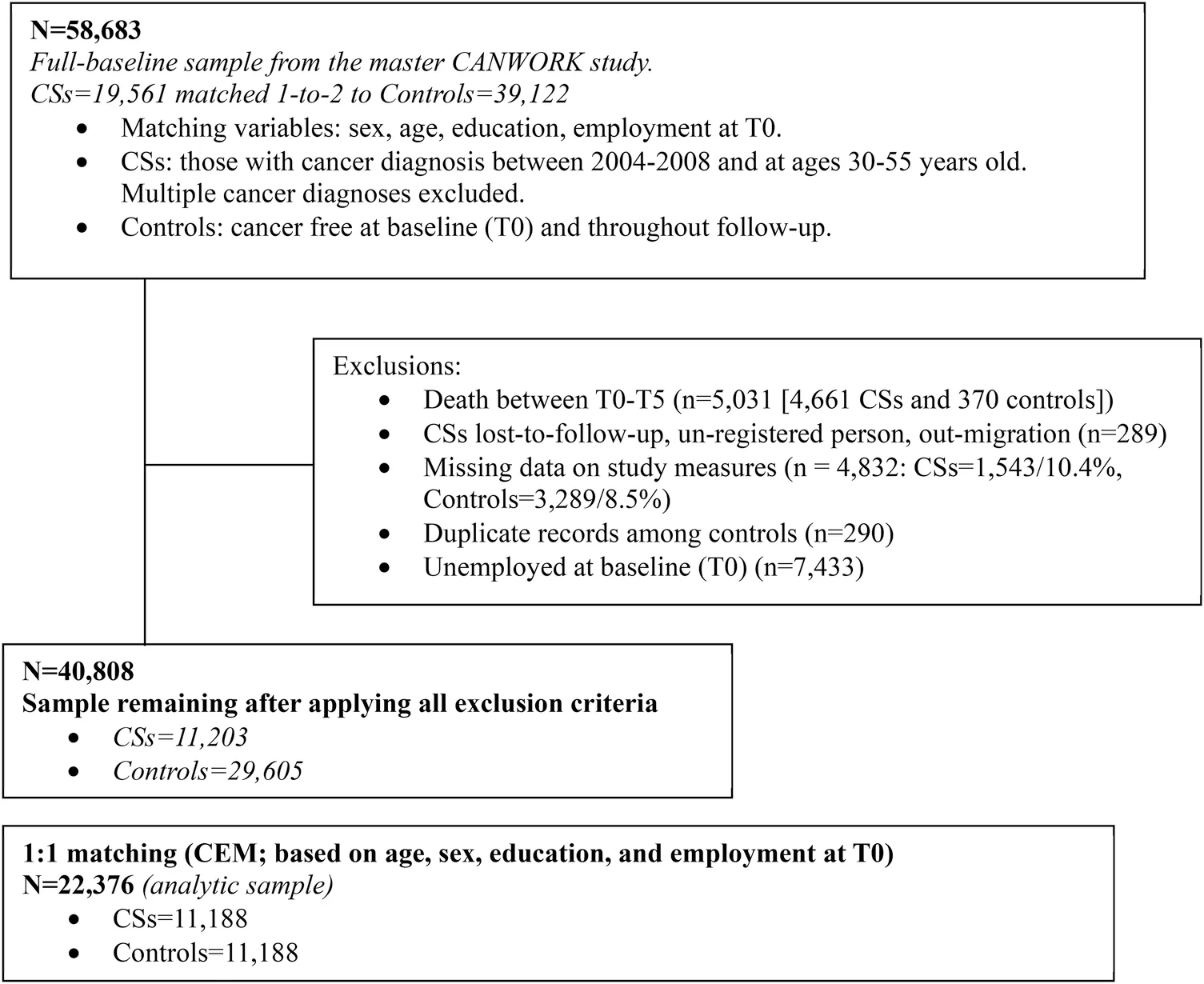
Flow diagram of the derivation of the analytic sample.

Due to the exclusion of individuals with missing data on the study measures, the original 1:2 matching of CSs and controls was not maintained. The remaining eligible CSs and controls were therefore rematched 1:1 using coarsened exact matching (CEM) (23), based on age (within 1-year bins), sex, education (4 levels), and baseline employment type (salaried/self-employed). The rematching was performed after individuals who died during the five-year follow-up had been excluded. The final matched sample comprised 22,376 individuals: 11,188 CSs and 11,188 controls.

CEM with the k2k option generated matched strata containing equal numbers of CSs and controls rather than separately identified one-to-one pairs. The conditional logistic regression analyses were therefore conditioned on the CEM strata. The final analytic sample comprised 22,376 individuals, including 11,188 CSs and 11,188 controls.

The study included individuals aged 30–55 years at cancer diagnosis, in accordance with the eligibility criteria of the broader CanWork project (21). The lower age limit was selected to focus on individuals who were more likely to have established labour-market attachment, whereas the upper limit ensured that all participants remained aged 60 years or younger at the five-year follow-up. Consequently, participants remained below Norway's ordinary retirement age of 67 years and the minimum age of 62 years for accessing certain early-retirement arrangements.

### Variables

Cancer status was coded as 1 for CSs and 0 for matched controls. The primary outcome was DP receipt five years after cancer diagnosis, based on information from Statistics Norway's FD-Trygd database. The outcome was coded as 1 if the individual was registered as receiving either partial or full DP at the five-year follow-up and 0 if the individual was not registered as receiving DP at that time. The latter category comprised all individuals who were not receiving DP at the five-year follow-up, irrespective of whether they were employed, unemployed, receiving sickness benefits, or had another labour-market status. The measure therefore represents DP receipt at the five-year follow-up point, regardless of when DP was first granted during the follow-up period. The analysis did not capture the timing or duration of DP receipt or transitions into or out of DP during the intervening years. Although all participants were employed at baseline, we also examined baseline DP receipt and confirmed that none were receiving partial or full DP at that time. The following variables were included as covariates: marital status, having children, income, immigrant background, and employment industry (see Table 1 for a more detailed description of how the variables are operationalized). For CSs specifically, we also included cancer type (based on ICD-10 codes) and cancer stage at diagnosis (local, regional, distant, unknown).

**Table 1:** Sample characteristics.

	Women		Men		All	
Characteristic	CSs	Control	CSs	Control	CSs	Control
	*N* = 6,959	*N* = 6,959	*N* = 4,229	*N* = 4,229	*N* = 11,188	*N* = 11,188
Sociodemographic variables						
Age at diagnosis, years, mean (SD)	46.22 (6.61)	46.22 (6.62)	45.65 (7.34)	45.66 (7.35)	46.00 (6.90)	46.01 (6.91)
*Education at diagnosis*						
Basic/unknown	1,235 (17.7)	1,235 (17.7)	713 (16.9)	713 (16.9)	1,948 (17.4)	1,948 (17.4)
Secondary	2,985 (42.9)	2,985 (42.9)	2,119 (50.1)	2,119 (50.1)	5,104 (45.6)	5,104 (45.6)
Lower university	2,265 (32.5)	2,265 (32.5)	926 (21.9)	926 (21.9)	3,191 (28.5)	3,191 (28.5)
Upper university	474 (6.8)	474 (6.8)	471 (11.1)	471 (11.1)	945 (8.4)	945 (8.4)
*Marital status at diagnosis*						
Married/cohabiting	4,233 (60.8)	4,278 (61.5)	2,563 (60.6)	2,491 (58.9)	6,796 (60.7)	6,769 (60.5)
Separated/divorced	1,206 (17.3)	1,260 (18.1)	520 (12.3)	545 (12.9)	1,726 (15.4)	1,805 (16.1)
Widowed	109 (1.6)	97 (1.4)	23 (0.5)	26 (0.6)	132 (1.2)	123 (1.1)
Single	1,411 (20.3)	1,324 (19.0)	1,123 (26.6)	1,167 (27.6)	2,534 (22.6)	2,491 (22.3)
*Children at diagnosis*						
No children	911 (13.1)	774 (11.1)	842 (19.9)	766 (18.1)	1,753 (15.7)	1,540 (13.8)
Youngest child aged 0–5	745 (10.7)	809 (11.6)	780 (18.4)	853 (20.2)	1,525 (13.6)	1,662 (14.9)
Youngest child aged 6–18	2,881 (41.4)	2,892 (41.6)	1,581 (37.4)	1,599 (37.8)	4,462 (39.9)	4,491 (40.1)
Youngest child aged 19+	2,422 (34.8)	2,484 (35.7)	1,026 (24.3)	1,011 (23.9)	3,448 (30.8)	3,495 (31.2)
*Income at diagnosis*						
First quartile	1,382 (19.9)	1,408 (20.2)	300 (7.1)	308 (7.3)	1,682 (15.0)	1,716 (15.3)
Second quartile	2,130 (30.6)	2,203 (31.7)	741 (17.5)	734 (17.4)	2,871 (25.7)	2,937 (26.3)
Third quartile	2,071 (29.8)	2,044 (29.4)	1,219 (28.8)	1,218 (28.8)	3,290 (29.4)	3,262 (29.2)
Fourth quartile	1,376 (19.8)	1,304 (18.7)	1,969 (46.6)	1,969 (46.6)	3,345 (29.9)	3,273 (29.3)
*Immigrant background at diagnosis*						
No immigrant background	6,590 (94.7)	6,543 (94.0)	4,006 (94.7)	3,972 (93.9)	10,596 (94.7)	10,515 (94.0)
Immigrant background	369 (5.3)	416 (6.0)	223 (5.3)	257 (6.1)	592 (5.3)	673 (6.0)
*Employment industry at diagnosis*						
Agriculture, forestry, fishing	70 (1.0)	90 (1.3)	139 (3.3)	150 (3.5)	209 (1.9)	240 (2.1)
Manufacturing	446 (6.4)	423 (6.1)	757 (17.9)	768 (18.2)	1,203 (10.8)	1,191 (10.6)
Construction	76 (1.1)	81 (1.2)	466 (11.0)	480 (11.4)	542 (4.8)	561 (5.0)
Wholesale/retail/auto repair	731 (10.5)	731 (10.5)	520 (12.3)	536 (12.7)	1,251 (11.2)	1,267 (11.3)
Transport, storage	264 (3.8)	268 (3.9)	426 (10.1)	383 (9.1)	690 (6.2)	651 (5.8)
Accommodation, food service	121 (1.7)	147 (2.1)	53 (1.3)	51 (1.2)	174 (1.6)	198 (1.8)
Admin, support services	487 (7.0)	493 (7.1)	478 (11.3)	469 (11.1)	965 (8.6)	962 (8.6)
Public admin, defence	551 (7.9)	575 (8.3)	317 (7.5)	305 (7.2)	868 (7.8)	880 (7.9)
Education	902 (13.0)	892 (12.8)	274 (6.5)	258 (6.1)	1,176 (10.5)	1,150 (10.3)
Human health, social work	2,487 (35.7)	2,491 (35.8)	272 (6.4)	280 (6.6)	2,759 (24.7)	2,771 (24.8)
Other industries	593 (8.5)	591 (8.5)	467 (11.0)	462 (10.9)	1,060 (9.5)	1,053 (9.4)
Unspecified industry	231 (3.3)	177 (2.5)	60 (1.4)	87 (2.1)	291 (2.6)	264 (2.4)
Clinical factors (CSs only)						
*Cancer stage at diagnosis*						
Local	3,671 (52.8)	–	1,996 (47.2)	–	5,667 (50.7)	–
Regional	1,582 (22.7)	–	647 (15.3)	–	2,229 (19.9)	–
Distant	180 (2.6)	–	100 (2.4)	–	280 (2.5)	–
Unknown	1,526 (21.9)	–	1,486 (35.1)	–	3,012 (26.9)	–
*Cancer type/diagnosis at diagnosis*						
Buccal cavity & pharynxd^a^	12 (0.2)	–	7 (0.2)	–	19 (0.2)	–
Digestive organs	428 (6.2)	–	515 (12.2)	–	943 (8.4)	–
Respiratory, intrathoracic organs	69 (1.0)	–	90 (2.1)	–	159 (1.4)	–
Bone, articular cartilage	6 (0.1)	–	8 (0.2)	–	14 (0.1)	–
Blood^b^	171 (2.5)	–	225 (5.3)	–	396 (3.5)	–
Skin^c^	862 (12.4)	–	592 (14.0)	–	1,454 (13.0)	–
Mesothelial, soft tissue	49 (0.7)	–	55 (1.3)	–	104 (0.9)	–
Breast	2,894 (41.6)	–	2 (0.0)	–	2,896 (25.9)	–
Female genital organs	993 (14.3)	–	0 (0.0)	–	993 (8.9)	–
Male genital organs	0 (0.0)	–	1,272 (30.1)	–	1,272 (11.4)	–
Urinary tract	128 (1.8)	–	348 (8.2)	–	476 (4.3)	–
Eye, brain & other CNS^d^	570 (8.2)	–	412 (9.7)	–	982 (8.8)	–
Thyroid, other endocrine glands	397 (5.7)	–	207 (4.9)	–	604 (5.4)	–
Ill-defined, secondary, unspecified sites	221 (3.2)	–	317 (7.5)	–	538 (4.8)	–
Unknown	159 (2.3)	–	179 (4.2)	–	338 (3.0)	–
DP^e^ *n* (%)	952 (13.7)	489 (7.0)	393 (9.3)	157 (3.7)	1,345 (12.0)	646 (5.8)

Values are *n* (%) unless otherwise indicated. There were no statistically significant differences between CSs and controls, except for variables ‘children’ (*χ*^2^ = 20.08, *p* < 0.001) and ‘immigrant background’ (*χ*^2^ = 5.50, *p* = 0.019).

a Lip, oral cavity, pharynx.

b Haematopoietic & reticuloendothelial tissues.

c Including malignant melanoma but not basal cell carcinomas.

d other CNS, other parts of the central nervous system.

e DP, receipt of partial or full disability pension at the five-year follow-up.

### Study measures

Marital status was recoded into a binary variable (0 = married or cohabiting, 1 = not married), and income was categorized into quartiles, with the “highest quartile” used as the reference group. Presence of children was categorized into four mutually exclusive groups (no children, children aged 0–5, 6–18, and 19+), with “no children” as the reference. Participants with children in more than one age group were therefore classified according to the age of their youngest child. Immigrant background was operationalized as a study-specific binary variable derived from a three-category nativity-status variable obtained from Statistics Norway: Norwegian native, first-generation immigrant, and second-generation immigrant with one or both parents classified as immigrants. For the present analyses, the first- and second-generation immigrant categories were combined (0 = no immigrant background [Norwegian native]; 1 = immigrant background [first- or second-generation immigrant]). Accordingly, the “immigrant background” category included both first-generation immigrants and individuals classified in the source data as second-generation immigrants, including individuals born in Norway to one or both immigrant parents. This study-specific binary grouping combines categories distinguished in the source data and should not be interpreted as equivalent to foreign-born or first-generation immigrant status. Unlike our related CanWork publication (22), which classified first-generation immigrants as Western or non-Western, the present binary classification combines first- and second-generation immigrants without distinguishing between immigrant generations or countries or regions of origin.

Education was initially classified into four categories: basic or unknown education, secondary education, lower university education, and upper university education. These categories were used in the matching procedure and are presented in Table 1. For the regression analysis among CSs presented in Table 3, lower and upper university education were combined into a single tertiary-education category, which served as the reference group. This recoding produced a more parsimonious model and ensured sufficient numbers in each educational category. Employment industry was included as a categorical variable with 12 categories according to the Norwegian Standard Industrial Classification (24). Sectors with relatively few participants were combined into a broad “other industries” category to avoid small cell sizes and unstable estimates. This category includes sectors such as finance, real estate, information technology, and arts. Participants whose industry information was assigned a valid registry code indicating an unspecified or unclassifiable industry were retained in an “unspecified industry” category rather than excluded from the analyses. This category was distinct from system-missing industry values, which contributed to the missing-data exclusions described above. Human health and social work was selected as the reference category because it was the largest employment-industry group in the sample and therefore provided a sufficiently large and stable comparison group.

In the analysis of CSs (Table 3), cancer type was classified according to ICD-10 codes. The combined category “ill-defined, secondary, and unspecified cancers” corresponded to ICD-10 codes C76–C80. It included malignant neoplasms of other or ill-defined sites (C76), secondary malignant neoplasms (C77–C79), and malignant neoplasms without specification of site (C80). This category was retained to allow these cases to remain in the analysis. However, it represents a heterogeneous registry category rather than a distinct clinical cancer type and should therefore be interpreted cautiously. In addition, cases assigned a valid registry code indicating an unknown cancer type were retained in an “unknown” category. These cases were not treated as system missing. Cancer stage at diagnosis was categorized as local, regional, distant, or unknown according to the Cancer Registry of Norway's summary-stage classification. This classification was selected because it provides a common measure of disease extent across different cancer sites. Local-stage disease was confined to the primary organ. Regional-stage disease involved invasion of neighbouring tissue outside the primary organ or spread to regional lymph nodes, whereas distant-stage disease involved spread to other organs or distant lymph nodes. The unknown category included cases in which the primary site was unknown or there was insufficient information to determine stage. For some cancer sites, patients who received neoadjuvant treatment were also classified as having unknown stage (25). The unknown-stage category represented a valid registry-coded classification; these cases were therefore retained in the analyses rather than treated as system-missing stage values. Digestive-organ cancers were used as the reference category because they represent one of the most common cancer groups among working-age CSs in our sample (approximately 8%–9%) and are well represented in both men and women. Localized cancer was selected as the reference category for cancer stage, as it reflects the earliest stage of disease.

### Statistical analysis

Descriptive statistics (Table 1) were computed using IBM SPSS Statistics version 29 to summarize the sample characteristics. Conditional logistic regression models were estimated in R version 4.5.0 using the survival package to examine the association between cancer status and receiving DP five years after diagnosis. Conditional logistic regression was used to account for the matched design, with the analyses conditioned on the CEM strata/stratum identifier. Two models were specified in Table 2. Model 1 included cancer status (CSs vs. controls) as the only predictor. Model 2 additionally adjusted for the sociodemographic covariates (e.g., marital status, income, having children). Age, sex, and education were not included in the models, as these variables were used in the matching procedure. Although age, sex, and education were controlled through matching, remaining sociodemographic and employment-related variables were included to account for residual heterogeneity within matched sets and to improve precision of the estimated associations. A separate subgroup analysis among CSs was conducted (Table 3) to examine the association between cancer type and cancer stage on DP receipt, using the same set of covariates as in the conditional logistic regression. Age, sex, and education were included in addition to the covariates included in the analyses presented in Table 2. In all analyses, statistical significance was defined as *p* < .05.

**Table 2:** Odds ratios (ORs) and 95% confidence intervals (CIs) for DP receipt five years post-diagnosis (*n* = 22,376), estimated using conditional logistic regression.

	Model 1	Model 2
Variable	OR (95%CI)	OR (95%CI)
Cancer status (Controls as reference group)	2.33 (2.10–2.57)***	2.37 (2.15–2.63)***
Marital status_T0 (married/cohabiting as reference group)		1.18 (1.06–1.31)**
Children_T0 (No children as reference group)		
Child age 0–5		0.50 (0.37–0.68)***
Child age 6–18		0.87 (0.74–1.03)
Child age 19+		0.99 (0.84–1.18)
Income_T0 (4th quartile as reference group)		
1st quartile		3.17 (2.64–3.81)***
2nd quartile		2.71 (2.29–3.21)***
3rd quartile		1.81 (1.53–2.15)***
Immigrant background at diagnosis (reference: no immigrant background)		0.89 (0.71–1.13)
Employment industry_T0 (“Human health and social work” as reference group)		
Agriculture, forestry, fishing		0.86 (0.59–1.25)
Manufacturing		0.83 (0.68–1.00)
Construction		0.83 (0.63–1.10)
Wholesale, retail; repair of auto/motorcycles		0.94 (0.79–1.11)
Transportation and storage		0.85 (0.67–1.07)
Accommodation and food service		0.89 (0.61–1.30)
Administrative and support service		0.68 (0.54–0.86)***
Public administration and defence		0.66 (0.53–0.82)***
Education		0.93 (0.77–1.12)
Other industries		0.88 (0.72–1.07)
Unspecified		2.46 (1.93–3.14)***

DP refers to receipt of partial or full disability pension at the five-year follow-up.

OR, odds ratio; CI, confidence interval; CSs, cancer survivors.

** *p* < .01.

*** *p* < .001.

Bold values indicates to the significant values at *p < .001 and *p* < .01.

**Table 3:** Odds ratios (ORs) and 95% confidence intervals (CIs) for DP receipt five years post-diagnosis among CSs (*n* = 11,188), estimated using logistic regression.

	Model 1	Model 2
Variable	OR (95%CI)	OR (95%CI)
Cancer type (digestive organs as reference group)		
Buccal cavity & pharynx^a^	1.05 (0.16–3.76)	1.38 (0.29–6.65)
Respiratory, intrathoracic organs	2.81 (1.86–4.20)***	2.70 (1.75–4.16)***
Bone, articular cartilage	2.42 (0.54–8.00)	3.99 (0.95–16.66)
Blood^b^	2.25 (1.57–3.21)***	3.13 (2.14–4.56)***
Skin^c^	0.52 (0.38–0.71)***	0.65 (0.47–0.91)*
Mesothelial, soft tissue	1.01 (0.49–1.88)	1.40 (0.70–2.80)
Breast	1.25 (1.00–1.57)	1.07 (0.84–1.38)
Female genital organs	1.07 (0.81–1.42)	0.96 (0.71–1.31)
Male genital organs	0.66 (0.49–0.89)**	1.08 (0.78–1.49)
Urinary tract	0.99 (0.69–1.43)	1.02 (0.70–1.50)
Eye, brain, and other CNS^d^	1.98 (1.51–2.61)***	2.75 (2.06–3.67)***
Thyroid, other endocrine glands	0.92 (0.65–1.28)	1.28 (0.90–1.83)
Ill-defined, secondary sites, unspecified cancers	1.35 (0.95–1.93)	1.81 (1.24–2.63)**
Unknown	1.22 (0.83–1.76)	1.31 (0.88–1.94)
Cancer stage (local stage as reference group)		
Regional	1.30 (1.11–1.53)**	1.44 (1.22–1.70)***
Distant	2.00 (1.43–2.75)***	2.04 (1.45–2.87)***
Unknown	1.00 (0.84–1.19)	1.03 (0.85–1.24)
Age		1.10 (1.09–1.12)***
Sex (female as reference group)		0.86 (0.71–1.04)
Education (tertiary as a reference group)		
Basic/unknown education		2.19 (1.80–2.65)***
Secondary education		1.51 (1.28–1.79)***
Marital status_T0 (married/cohabiting as reference group)		1.20 (1.05–1.37)**
Children_T0 (No children as reference group)		
Child age 0–5		0.62 (0.44–0.88)**
Child age 6–18		1.01 (0.83–1.24)
Child age 19+		1.06 (0.86–1.30)
Income_T0 (4th quartile as reference group)		
1st quartile		2.98 (2.39–3.71)***
2nd quartile		2.77 (2.27–3.37)***
3rd quartile		1.88 (1.55–2.29)***
Immigrant background at diagnosis (reference: no immigrant background)		0.97 (0.73–1.28)
Employment industry (“Human health & social work” as reference group)		
Agriculture, forestry, fishing		0.98 (0.64–1.51)
Manufacturing		0.75 (0.59–0.95)*
Construction		0.75 (0.53–1.06)
Wholesale, retail; repair of auto/motorcycles		0.98 (0.80–1.21)
Transportation and storage		0.90 (0.68–1.20)
Accommodation and food service		0.85 (0.52–1.38)
Administrative and support service		0.69 (0.53–0.90)**
Public administration and defence		0.82 (0.63–1.08)
Education		0.85 (0.67 –1.08)
Other industries		0.90 (0.70–1.15)
Unspecified		2.18 (1.61–2.96)***
Nagelkerke *R*^2^	0.036	0.175

The outcome was receipt of partial or full disability pension at the five-year follow-up.

OR, odds ratio; CI, confidence interval; CSs, cancer survivors.

«a» Lip, oral cavity, pharynx. «b» Haematopoietic & reticuloendothelial tissues. «c» Including malignant melanoma but not basal cell carcinomas. «d» other CNS = other parts of the central nervous system.

* *p* < .05.

** *p* < .01.

*** *p* < .001.

Bold values indicates to the significant values at *p < .001 and *p* < .01.

### Ethical consideration

The study was approved by the Norwegian Data Protection Authority (reference 16/00235), the Norwegian Social Science Data Services (NSD), and the Regional Committee for Medical and Health Research Ethics, Mid-Norway (REK Midt, reference 2016/830). All data were de-identified and analyzed in accordance with national regulations on registry-based research. As the study used secondary data from Norwegian national registries, informed consent from individual participants was not required.

## Results

### Sample characteristics

The final analytic sample comprised 22,376 individuals, including 11,188 CSs and 11,188 matched cancer-free controls. Women represented 62.2% of the sample, and the mean age at baseline was approximately 46 years. All participants were employed at baseline. Approximately 61% were married or cohabiting, and 5.7% were classified as having an immigrant background. As expected from the matching procedure, CSs and controls had similar distributions of age, sex, education, and baseline employment type, and most other sociodemographic and employment characteristics. Further sample characteristics are presented in Table 1. However, two variables differed statistically between CSs and controls. Compared with controls, CSs were more likely to have no children (15.7% vs. 13.8%; *χ*^2^ = 20.08, *p* < 0.001) and slightly less likely to be classified as having an immigrant background (5.3% vs. 6.0%; *χ*^2^ = 5.50, *p* = 0.019). Among CSs, most had local-stage disease at diagnosis, followed by regional-stage disease, while distant-stage disease was least common.

Five years after diagnosis, 1,345 of 11,188 CSs (12.0%) and 646 of 11,188 controls (5.8%) were receiving DP, corresponding to an absolute difference of 6.2 percentage points.

### Difference between CSs and controls in receiving DP

Table 2 presents the results from the conditional logistic regression models examining DP receipt five years after diagnosis. In model 1 which included cancer status as the only predictor, CSs had more than twice the odds of receiving DP compared with controls (OR = 2.33, 95% CI: 2.10–2.57). In Model 2, after additional adjustment for sociodemographic and employment-related variables, the estimate remained largely unchanged (OR = 2.37, 95% CI: 2.15–2.63).

For CSs and controls: Not being married was associated with higher odds of DP receipt (OR = 1.18, 95% CI: 1.06–1.31). A strong income gradient was observed: individuals in the lowest income quartile had more than three times the odds of receiving DP compared with those in the highest income quartile (OR = 3.17, 95% CI: 2.64–3.81).

Having children aged 0–5 years, compared with having no children was associated with lower odds of receiving DP (OR = 0.50, 95% CI: 0.37–0.68). Immigrant background was not significantly associated with the odds of receiving DP (OR = 0.89, 95% CI: 0.71–1.13).

In terms of employment industry (reference: “Human health and social work”), employment in administrative and support services (OR = 0.68, 95% CI: 0.54–0.86) and public administration and defence (OR = 0.66, 95% CI: 0.53–0.82) was associated with lower odds of DP. Individuals in unspecified industries had substantially higher odds of receiving DP (OR = 2.46, 95% CI: 1.93–3.14).

### Further analysis: cancer type and cancer stage

Table 3 shows the logistic regression results among CSs only. Using digestive organ cancers as the reference, several cancer types were associated with higher odds of receiving DP in Model 1, including respiratory/intrathoracic (OR = 2.81, 95% CI: 1.86–4.20), blood cancers (OR = 2.25, 95% CI: 1.57–3.21), and eye, brain, and other CNS cancers (OR = 1.98, 95% CI: 1.51–2.61). By contrast, skin cancers (OR = 0.52, 95% CI: 0.38–0.71) and male genital cancers (OR = 0.66, 95% CI: 0.49–0.89) were associated with lower odds of receiving DP than digestive-organ cancers.

In Model 2, after additional adjustment for sociodemographic and employment-related variables, respiratory/intrathoracic cancers (OR = 2.70, 95% CI: 1.75–4.16), blood cancers (OR = 3.13, 95% CI: 2.14–4.56), and eye, brain, and other CNS cancers (OR = 2.75, 95% CI: 2.06–3.67) remained associated with higher odds of receiving DP than digestive-organ cancers. The combined category of Ill-defined, secondary, and unspecified cancers was also associated with higher odds of receiving DP than digestive-organ cancers (OR = 1.81, 95% CI: 1.24–2.63). Skin cancer was associated with lower odds of receiving DP than digestive-organ cancers (OR = 0.65, 95% CI: 0.47–0.91). By contrast, the association between male genital cancers and receiving DP observed in model 1 was no longer statistically significant in the fully adjusted model 2.

Regarding cancer stage in model 2, both regional (OR = 1.44, 95% CI: 1.22–1.70) and distant-stage cancers (OR = 2.04, 95% CI: 1.45–2.87) were associated with higher odds of receiving DP compared to localized cancers.

Among sociodemographic factors, each additional year of age was associated with approximately 10% higher odds of receiving DP (OR = 1.10, 95% CI: 1.09–1.12). Compared to tertiary education, CSs with basic/unknown education (OR = 2.19, 95% CI: 1.80–2.65) and secondary education (OR = 1.51, 95% CI: 1.28–1.79) had higher odds of receiving DP. Being unmarried was associated with higher odds of receiving DP (OR = 1.20, 95% CI: 1.05–1.37). Individuals in the lowest income quartile had nearly three times the odds of receiving DP compared with those in the highest income quartile (OR = 2.98, 95% CI: 2.39–3.71). Having children aged 0–5 was associated with lower odds of receiving DP (OR = 0.62, 95% CI: 0.44–0.88). Immigrant background was not associated with receiving DP (OR = 0.97, 95% CI: 0.73–1.28). Compared with employment in human health and social work sectors, employment in unspecified industries was associated with higher odds of receiving DP (OR = 2.18, 95% CI: 1.61–2.96), whereas employment in manufacturing (OR = 0.75, 95% CI: 0.59–0.95) and in administrative and support services (OR = 0.69, 95% CI: 0.53–0.90) was associated with lower odds of receiving DP.

Model fit improved substantially from the clinical model (Model 1) to the fully adjusted model (Model 2), with Nagelkerke *R*^2^ increasing from 0.036 to 0.175 following the addition of sociodemographic and employment-related variables.

## Discussion

In this nationwide matched cohort study, CSs had more than twice the odds of receiving DP five years after diagnosis compared with cancer-free controls. By including survivors of multiple cancer sites within a single analytical framework, our study enables a comparison of receiving DP across cancer types in a Norwegian context. This broader perspective complements earlier single-site studies and contributes to a more comprehensive understanding of DP receipt among CSs. The findings are consistent with previous Nordic studies showing higher odds of receiving DP among CSs (5, 7, 8, 26). Such consistency highlights that the associations between cancer diagnosis and DP receipt remain a persistent public health concern across relatively generous welfare states in the Nordic region.

At the five-year follow-up, the absolute difference in DP receipt between CSs and controls was 6.2 percentage points. Given the large sample, statistically significant associations of modest magnitude should not automatically be interpreted as clinically or practically important.

Consistent with previous research (8, 27), we found that cancers with a poor prognosis or intensive treatment (e.g., lung, blood, eye, brain, and other CNS cancers) were associated with higher odds of receiving DP among working-age CSs. In contrast, cancers with more favorable prognoses such as skin cancers (28), were associated with lower odds of DP. These site-specific disparities align with findings from previous Nordic studies (8, 27). Blood cancers often require dose-intensive chemotherapy or hematopoietic stem cell transplantation, which may involve prolonged immune suppression, peripheral neuropathy, and late infections. These factors have been associated with higher odds of DP receipt (29). Similarly, lung CSs frequently experience fatigue, pain, and chronic pulmonary impairments that have been associated with limited daily functioning and lower work ability (30, 31). CNS tumors and their treatment have been associated with lasting neurological or cognitive impairments (32). The combined category of ill-defined, secondary, and unspecified cancers was associated with higher odds of DP receipt than digestive-organ cancers. However, as noted in the Methods, this category reflects diagnostic and coding uncertainty rather than a shared biological or prognostic profile. The association should therefore not be attributed to a particular disease mechanism or prognosis. In contrast, skin cancers often have favorable survival and less disabling treatments, which likely facilitates the better work participation and subsequently lower odds of DP receipt (28). Overall, our findings support the consensus that cancer type is strongly associated with DP receipt in CSs.

In addition, consistent with earlier research from Nordic countries, cancer stage was strongly associated with DP receipt (5–7). Cancers diagnosed at regional or distant stages had substantially higher odds of receiving DP than those with localized stage. This likely reflects greater functional impairments and pain associated with more advanced stages (33, 34). These findings underscore the importance of early detection and targeted support for CSs with advanced-stage diagnoses, which may help lower the odds of DP receipt.

Beyond clinical characteristics, we observed sociodemographic disparities in DP receipt among CSs. Lower income was strongly and monotonically associated with DP receipt. CSs in the lowest income quartile had the highest odds of receiving DP, whereas those in the highest quartile had the lowest. This is in line with Carlsen et al. (16), who reported that lower income was associated with higher odds of DP receipt. One explanation could be that lower-income individuals are more often in physically demanding jobs with limited flexibility or accommodations. This may make continued work harder when health is compromised.

We found that having very young children (ages 0–5) was associated with lower odds of receiving DP. This finding is consistent with Hauglann et al. (6), who reported higher odds of DP among individuals without children under 18 in the household. Similar patterns have been documented in Nordic public health research. Karlsson et al. (35) observed that, among individuals on long-term sick leave in Sweden, having young children was associated with a lower probability of receiving DP. Furthermore, this finding aligns with the Norwegian cohort study by Gjesdal and Bratberg (36), which reported that having children under 7 years was associated with lower odds of DP receipt. Together, these findings suggest that having young children was associated with lower odds of DP receipt. This may partly reflect that individuals who are healthy enough to care for small children are also better able to remain in the workforce. In a Nordic welfare context, the availability of publicly funded childcare and kindergarten services, as well as informal childcare support such as grandparental care, may further facilitate continued employment among parents with cancer who have very young children. This may, in turn, lower the odds of receiving DP. However, the association may reflect unmeasured differences between CSs with young children and those with no children or older children and should not be interpreted as evidence of a protective causal effect. In contrast to earlier Nordic studies reporting higher DP receipt among foreign-born individuals (7, 17, 19), we found no significant association between immigrant background and receiving DP. In the present study, all participants were employed at the time of cancer diagnosis, which should be taken into account when interpreting these findings. Future studies including individuals with more heterogeneous employment situations at diagnosis are needed to further examine potential differences in DP receipt by immigrant background.

Our findings also indicate meaningful differences in DP receipt across employment industries. Compared with individuals working in the human health and social work sector, those in administrative and support services had significantly lower odds of receiving DP. This pattern was observed both in the full sample and among CSs specifically. Among CSs specifically, manufacturing was also associated with lower odds of receiving DP. These findings call for future research, as we currently lack a clear explanation for why employees in these sectors appear to have more favourable outcomes. By contrast, individuals classified in ‘unspecified industries’ had higher odds of receiving DP. This category represents a heterogeneous group whose employment industry was assigned a valid registry code indicating an unspecified or unclassifiable industry. The higher odds of receiving DP may therefore reflect a range of underlying employment situations that cannot be clearly distinguished in the present data. These situations may be associated with more limited access to workplace accommodations, lower job security, or more variable income sources. However, such mechanisms cannot be examined directly in this study. Similarly, the ‘other industries’ category combined several smaller industry groups that did not fit elsewhere in the classification and showed no clear association with DP receipt. Because these industries likely differ considerably in their working conditions and demands, associations in opposite directions may have offset one another. Therefore, the overall null finding does not rule out the possibility that individual industries within this category were associated with either higher or lower odds of DP receipt.

Our findings should be interpreted in light of several limitations. First, the study relied on registry data and therefore lacked detailed clinical and treatment information. Psychosocial measures, such as comorbidity, cognitive burden, fatigue, or mental health status were also unavailable. The absence of these variables means that important prognostic factors were not adjusted for. Second, some baseline characteristics (e.g., income and marital status) were measured only at the time of cancer diagnosis. Third, our cohort included only relatively young individuals who were employed at diagnosis and survived throughout the five-year follow-up. Consequently, the findings may not be generalisable to CSs with aggressive disease courses who died before the five-year follow-up, individuals outside the studied age range (including older working-age survivors) or those who were not employed at diagnosis. The available data permitted a uniform follow-up of five years, and receipt of partial or full DP was assessed only at the five-year endpoint. Consequently, we could not examine longer-term changes in DP receipt or capture the duration of partial or full DP receipt. Individuals who received DP earlier but were no longer receiving it at five years were not counted as recipients at the endpoint. This may lead to an underestimation of total DP receipt in both groups. At later follow-up points, DP receipt may also reflect health conditions associated with ageing, making the specific association with cancer more difficult to distinguish. Fourth, we were unable to determine the specific medical reason for the DP receipt. Although the study focused on CSs, some individuals may have received DP due to other medical conditions unrelated to their cancer diagnosis. However, all participants were employed and not receiving DP at the time of diagnosis, meaning that any DP received during follow-up occurred after cancer diagnosis. Combined with the well-established long-term health consequences of cancer and its treatment, this makes it plausible that cancer and its long-term consequences contributed to DP receipt in many of these cases. Prospective cohort studies that collect more comprehensive clinical and psychosocial data (including treatment, functional capacity, and workplace support) would enable a more detailed examination of factors associated with DP receipt among CSs. Fifth, the study follow-up preceded the 2015 reform of the Norwegian DP system. The findings therefore reflect the regulations in place during the study period and may not be directly generalizable to CSs diagnosed under the current policy framework. Sixth, 4,832 individuals were excluded because of system-missing values on one or more study measures. The proportion excluded because of missing data was somewhat higher among CSs than controls (10.4% vs. 8.5%). If individuals with missing data differed systematically from those retained in the analytic sample, this exclusion may have introduced selection bias and affected the comparison between CSs and controls. Finally, our findings are situated within Norway's universal, publicly funded welfare system, which offers comparatively generous income replacement relative to many other countries. We reason that in welfare systems with more restrictive DP eligibility, the relationship between cancer diagnosis and DP receipt may differ substantially. Caution is therefore needed in generalizing these findings beyond Nordic (or similarly structured welfare) contexts.

## Conclusion

This nationwide matched cohort study shows that CSs aged 30–55 and employed at diagnosis had higher odds of receiving DP five years later than matched cancer-free controls in Norway. Among CSs, cancers with poorer prognoses or intensive treatment needs (such as respiratory, blood, and CNS cancers), as well as advanced-stage disease, were associated with higher odds of DP receipt. In contrast, skin cancers were associated with lower odds of DP receipt. Sociodemographic factors, including older age, lower education, lower income, being unmarried, and employment in unspecified industries, were also associated with higher odds of DP receipt. Having young children was associated with lower odds of DP receipt. These findings suggest that tailored occupational rehabilitation and work-retention strategies addressing both clinical and socioeconomic circumstances may be valuable for CSs. Such support may be particularly relevant for survivors with advanced-stage disease, cancer types associated with poorer prognosis or intensive treatment needs (e.g., respiratory, blood, or CNS cancers), or with lower income or education.

## Data Availability

The data analyzed in this study is subject to the following licenses/restrictions: only approved researchers can have access to the dataset. Requests to access these datasets should be directed to shahram.moradi@usn.no.
